# Supplementation Strategies to Reduce Muscle Damage and Improve Recovery Following Exercise in Females: A Systematic Review

**DOI:** 10.3390/sports4040051

**Published:** 2016-11-11

**Authors:** Jessica L. Köhne, Michael J. Ormsbee, Andrew J. McKune

**Affiliations:** 1 Discipline of Biokinetics, Exercise and Leisure Sciences, School of Health Sciences, University of KwaZulu-Natal, Durban 3629, South Africa; kohnejessica@gmail.com; 2 Department of Nutrition, Food and Exercise Sciences, Institute of Sport Sciences and Medicine, Florida State University, Tallahassee, FL 32308, USA; mormsbee@fsu.edu; 3 Discipline of Sport and Exercise Science, University of Canberra Research Institute for Sport and Exercise, Faculty of Health, University of Canberra, Canberra ACT 2601, Australia

**Keywords:** EIMD, recovery, protein, blueberries, inflammation

## Abstract

Exercise-induced muscle damage (EIMD) caused by unaccustomed or strenuous exercise can result in reduced muscle force, increased muscle soreness, increased intramuscular proteins in the blood, and reduced performance. Pre- and post-exercise optimal nutritional intake is important to assist with muscle-damage repair and reconditioning to allow for an accelerated recovery. The increased demand for training and competing on consecutive days has led to a variety of intervention strategies being used to reduce the negative effects of EIMD. Nutritional intervention strategies are largely tested on male participants, and few report on sex-related differences relating to the effects of the interventions employed. This review focuses on nutritional intervention strategies employed to negate the effects of EIMD, focussing solely on females.

## 1. Introduction

Unaccustomed, eccentric exercise is known to result in exercise-induced muscle damage (EIMD), resulting in disruption and/or degradation of structural proteins in the muscle fibres as well as in connective tissue [1,2,3,4,5], with initial damage evident immediately post-exercise, involving the infiltration of inflammatory cells to the site of injury [6]. A level of muscle discomfort related to the EIMD, otherwise known as delayed onset of muscle soreness (DOMS), increases until subsiding five to seven days post-exercise [7]. Furthermore, losses in muscle strength, impaired muscle function, imbalances in muscle protein breakdown and protein synthesis have been noted with EIMD [2,4,8,9,10,11,12].

Due to the increasing training demands on athletes, optimizing as well as accelerating the recovery process is essential to enhance their performance [13,14]. Several intervention strategies have been proposed to negate the negative effects coupled with EIMD. These include interventions such as nutrition, pharmacological strategies, electrotherapeutic (nerve stimulation) and manual strategies (stretching, massage), cryotherapy and active exercise [13,15,16]. 

Numerous studies have examined the effects of nutritional supplementation interventions to reduce the effects of EIMD by increasing the net protein balance including whey protein isolate [10,17], branched-chain amino acids (BCAAs: leucine, isoleucine, and valine) [18,19,20], protein hydrolysates [21], leucine only [1], and creatine [22]; increasing anti-inflammatory and anti-oxidant activity through tart cherry juice [23,24] and blueberry [25]; as well as enhancing recovery through the consumption of chocolate milk [26,27], cow’s milk [5], and multi-ingredient supplements [28] to name a few. 

Although it has been reported that males and females may show similar strength and force decrements associated with EIMD, it has been noted that females tend to require longer periods of recovery when compared to males; however, reasons for this are unclear [29]. On the other hand, in a recent study, it was indicated that females are less fatigable than males and subsequently have a faster recovery [30]. In previous studies it was hypothesized that several of the sex-related differences are attributed to the female sex hormone 17β-estradiol [31,32,33]. It has been reported that oestrogen has the ability to influence skeletal muscle growth, gene expression, metabolism, contraction characteristics, and maintain muscle mass [34,35]. Oestrogen also enhances lipid oxidation and consequently down-regulates the utilization of carbohydrates both at rest and during sub-maximal exercise, thus affecting muscle metabolism [36]. In addition, oestrogen appears to have a protective effect with regards to DOMS compared with males following the same exercise protocol [30,37]. Finally, it has been reported that increases in oestrogen results in significant decreases in serum CK activities post-exercise or post-injury [33,38,39,40,41], in addition to attenuating circulating IL-6 levels dependent on menstrual cycle phase and oral contraceptive usage [33].

The majority of studies that have investigated the ergogenic effects of nutritional supplementation on exercise performance and recovery have focused predominantly on males [1,20,42,43,44,45]. However, a number of studies have focused on female rats and other animals [46,47,48], whilst several studies have focused on women [34,49,50,51,52,53,54,55,56]. The aim of this review is to examine the effects of dietary modalities on markers of EIMD and recovery in females following a bout of exercise.

## 2. Materials and Methods

### 2.1. Literature Review and Study Selection

The published literature was searched using the databases of PUBMED, SPORTDiscus, GoogleScholar and ScienceDirect in June 2016, for articles during the period 2005 to 2016. A variety of combinations of words and terms were entered for the search including, but not limited to: ‘females’, ‘exercise-induced muscle damage’, ‘recovery interventions’, ‘dietary interventions’, ‘supplement interventions’, ‘muscles’, ‘placebo controlled’, ‘exercise’, ‘training’, and ‘physical activity’. Hand searches of journal articles and of the reference lists from relevant publications were also performed to ensure, as far as practically possible, that all appropriate studies were considered for inclusion. Once all relevant articles had been located, the researcher individually considered each article for its appropriateness for inclusion based on the pre-determined inclusion criteria discussed below. Where there was uncertainty with regards to inclusion, discussion with a second researcher ruled on the inclusion or exclusion of the article. Several other desirable characteristics of studies were also discussed, such as the homogeneity of the participant population and the availability of information on the reliability of the exercise tests and measures employed, however, the inclusion criteria remained as they were.

### 2.2. Inclusion/Exclusion Criteria

**Figure 1:**
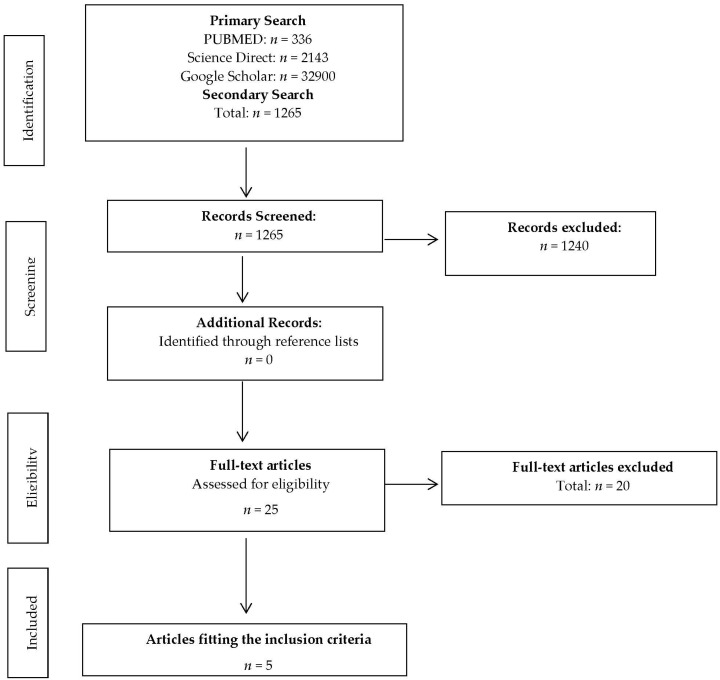
Schematic representation of the flow of information during the different phases of the systematic review.

*Design*. This analysis was limited to manuscripts using human participants in either a double blind or balanced, randomized designs, published in English-language peer-reviewed journals, and which had dietary or supplementation interventions. Summary details of the studies included in the review are listed in Table 1. Due to these inclusion criteria, several studies were excluded from the analysis, for a multitude of reasons. These included those with no female participants, used a cross-over study design with an insufficient wash out period (<30 days [57]), not published in peer-reviewed journals, or the many experiments that seemed only to be available as abstracts (Figure 1).

*Participants*. Studies with recreationally physically active or trained females between ages of 18 years and 40 years were included. Studies that examined both males and females were excluded, as well as studies only looking at males. 

*Outcome Measures*. Studies were included if muscular strength, pain perceptions, blood markers of muscle damage, and flexibility were measured following a supplementation intervention period and a bout of exercise-inducing muscle damage. Morphological outcomes considered were body weight (kg), body mass index (BMI; kg/m^2^), lean body mass (kg), body fat, circumferences (calf, thigh, arm, waist, chest, and hip), percent body fat, and waist to hip ratio (WHR). Inflammatory, indirect muscle damage indicators considered were creatine kinase (CK), C-reactive Protein (CRP), reactive oxygen species (ROS)/free radicals, myoglobin, lactate dehydrogenase (LDH), alpha-actinin, interferon (IFN)-γ and cytokines (IL-1α, IL-1β, IL-6, IL-8, IL-10, TNF-α). Additional blood markers included, but were not limited to, markers for anti-oxidant activity (reduced glutathione (GSH) and oxidized glutathione (GSSG)), and protein carbonyls. Furthermore, studies examining the use of visual analogue scale (VAS) measurements to subjectively rate muscle soreness were included. Strength and performance measures considered for this review included but were not limited to isometric strength testing, counter-movement jumps, squat jumps, and flexibility (sit-and-reach; cm).

## 3. Results and Discussion

### 3.1. Study Characteristics

Five published investigations met the inclusion criteria (see Table 1 for study details). The number of participants in each study ranged from 10 to 18. Study participants were females between the ages of 18 and 40 years. All participants were physically active, with one study including endurance trained athletes, two studies included recreationally active individuals, and two studies including NCAA Division III Basketball players.

Supplementation interventions used in the studies selected included whey protein vs. casein protein [58]; whey protein vs. soy protein [59]; carbohydrate (CHO) vs. CHO with protein vs. placebo [60]; chocolate milk (CM) vs. a placebo [61]; whey protein vs. maltodextrin (MD) [62]; and blueberry vs. control/placebo [25]. Supplementation ingestion protocols are summarized in Table 1. Two studies required participants to ingest the supplementation beverages both prior to and following the training/exercise sessions [58,62]; two studies required ingestion following muscle damaging exercise [25,60]; and one study required ingestion once a day, with no specified time point [59]. 

Three different exercise modes were used in the studies to assess the influence of a supplementation intervention, including resistance training [58,62], maximal eccentric contractions [25], and running [59,60,61]. Performance measures used included: (1) 1-repetition maximum (1-RM) measures of bench press and leg press [58,62]; (2) broad jump and vertical jump [58,62]; (3) Isometric testing [25,60]; (4) concentric and eccentric torque [25]; and (5) 5-10-5 shuttle runs [58,62]. Further measures used to assess the effects of the supplementation intervention included: (1) blood draws for markers of inflammation and/or muscle damage [25,59,60]; (2) perceptions of DOMS [25,60]; and (3) body composition [58,59].

### 3.2. Effects of the Supplementation Intervention on Indicators of Muscle Damage and Inflammation

#### 3.2.1. Perceptions of Muscle Damage

Participant perception of DOMS was established in two studies. One study used a visual analogue scale (VAS), with a scale from 0 to 100 mm [60], and the other study used a subjective rating of muscle soreness, rating from 0 (no pain) to 10 (very, very painful) [25].

Green, et al. [60] indicated no differences between groups following supplementation of either carbohydrate only (CHO) or carbohydrate with protein (CHO with Protein) or placebo; however, it was noted that, combined across all groups, the amount of muscle soreness peaked two days post-exercise, and then dissipated but remained significantly elevated three days post-exercise (*p* < 0.05) compared to pre-values. 

In a study comparing the consumption of New Zealand blueberries with a control, participants were required to rate their perceived soreness following stepping up and back down from a 40 cm box. The rating of perceived soreness (RPS) was only noted following 300 maximal eccentric repetitions; as such comparisons with ratings prior to the exercise cannot be made. McLeay, et al. [25] noted that there were significant differences between subjects (*p* < 0.001), however no difference was observed between the blueberry and control conditions (*p* = 0.723), nor was an interaction effect noted between time and treatment (*p* = 0.425). Furthermore, a significant time effect was noted (*p* < 0.001), with no significant treatment and group interaction effects were noted (*p* > 005).

The perceived soreness experienced following muscle damaging exercise was not attenuated by supplementation of either CHO, CHO with protein, or a blueberry beverage. However, the muscle soreness experienced appeared to have peaked two days following the muscle damaging exercise. 

#### 3.2.2. Strength Testing

In the studies which used strength testing measures (1-RM, maximal isometric strength, or concentric and eccentric torque), to aid in determining the effects of a supplementation intervention, one study recruited female recreational athletes [60], another recruited healthy, physically active resistance trained females [25], and the third and fourth studies NCAA Division III female basketball players [58,62]. The participants in all four studies participated in resistance training and/or aerobic training prior to their participation in the study.

In the study conducted by Green, et al. [60], isometric strength testing of the quadriceps in female runners was utilized prior to and following a bout of intermittent downhill running, to identify strength changes with supplementation. Similar torque values were noted for all groups (CHO, CHO with protein and placebo) at baseline (*p* = 0.27), as well as recovery of maximum isometric quadriceps strength not differing between groups at any time point (*p* = 0.21). However, it was noted that the maximum isometric quadriceps strength was reduced by 20.6% ± 1.5% immediately following the downhill run in all groups, and showed no significant improvement one-day post downhill run. Two days post downhill run, the maximal isometric strength showed a significant degree of recovery, however, was still reduced by 11.3% ± 2.3% from baseline values (*p* < 0.05). By three days post downhill run, all participants had recovered back to baseline strength values (−4.4% ± 1.5%), with no differences between treatment groups.

Although significant differences were noted between time points (*p* < 0.001) in the percentage change from pre-damage measures in peak isometric tension, peak and average concentric torque, and eccentric torque in the quadriceps muscle, values did not differ between the conditions; however, a significant treatment x time interaction effect was noted for the peak isometric tension (*p* = 0.047) [25]. The muscle damage experienced in the blueberry treatment group, elicited a decrease in peak torque by 20%, 24% and 21% for isometric tension, concentric and eccentric torque respectively, and the control by 17%, 28% and 20% respectively at 12 h post-exercise compared to pre. A similar decrease was seen in the average peak torque/tension for the blueberry (16%, 24% and 16%) and for the control (17%, 20%, and 24%) for isometric, concentric and eccentric respectively 12 h post. Furthermore, it was noted that by 60 h post-damaging exercise, the muscle function performance returned to pre-damaging values in both the blueberry and control groups. A faster rate of recovery was noted in the first 36 h post-damage in the blueberry treatment, with a significant interaction seen between time and treatment for peak isometric tension (*p* = 0.047). However, although improvements in performance at 36 h for the peak concentric and eccentric torque with blueberries compared to the control condition were evident, no significant interaction effect was noted between time and treatment (*p* = 0.564 and 0.578 respectively). Equivalent trends were observed with the average isometric tension, concentric and eccentric torque, again with no significant interaction between time and treatment (*p* = 0.597, 0.449 and 0.880 respectively).

Interestingly Wilborn, et al. [58] reported significant strength gains in 1-RM leg press (whey protein group: 88.7 ± 43.9 kg; casein protein group: 90.0 ± 48.5 kg, *p* < 0.001) and 1-RM bench press (whey protein group: 7.5 ± 4.6 kg; casein protein group: 4.3 ± 4.5 kg, *p* = 0.01) for both the casein protein and whey protein groups following a resistance training protocol. Similar trends were noted by Taylor, et al. [62], in which both WP (*p* < 0.001) and MD (*p* = 0.012) groups elicited strength gains in 1-RM bench press from baseline to follow-up (following the eight-week protocol). The whey protein group gained significantly more strength at the follow-up (+4.5 kg) when compared to the MD group (+2.3 kg; *p* = 0.04). Furthermore, both groups showed a 30 kg increase in 1-RM leg press following the eight-week protocol (*p* < 0.001) [62]. Improvements in vertical jump height (whey protein group: 4.1 ± 1.8 cm; casein protein group: 3.5 ± 6 cm, *p* < 0.001) and broad jump distance (whey protein group: 10.4 ± 6.6 cm; casein protein group: 12.9 ± 7.1 cm, *p* < 0.001), with agility performance improvements in both groups (*p* < 0.001) noted by Wilborn, et al. [58]. Taylor, et al. [62] indicated similar results: vertical jump improved in both groups (WP: *p* < 0.001; MD: *p* = 0.034), as well as broad jump performance improving by 7.2 cm following the eight-week program (*p* = 0.008). Agility performance was significantly improved in the WP group (*p* = 0.001) following the eight-week protocol, while no improvement was noted for the MD group.

Supplementation with whey [58,62] and casein [58] elicited greater improvements in strength and other performance indicators, while supplementation with CHO or CHO with protein, or blueberries, did not exhibit any difference between groups. It could thus be suggested that whey protein should be recommended for strength improvements.

#### 3.2.3. Markers of Muscle Damage and Inflammation

The results obtained from the studies which examined the effects of a supplementation intervention on markers of muscle damage and inflammation can be seen in Table 2 All of the studies indicated increases in markers of muscle damage following the exercise and supplementation interventions. Green et al. [60] examined serum creatine kinase (CK) activity in female athletes, and found that CK activity increased immediately post-exercise, peaking at one day post downhill run, and began decreasing on day two and three post-exercise, with no differences between treatment groups (*p* = 0.59). It was further noted that although the difference was not statistically significant, the CHO group experienced a greater increase in CK activity one day post downhill run, when compared with the CHO/protein group and placebo group. 

Another study that solely recruited female athletes [59], did not measure CK activity to identify the extent of muscle damage and inflammation, but rather studied a variety of cytokines (IL-1α, IL-1β, IL-2, IL-6, IL-8, TNF-α, IFN-γ) as well as C-reactive protein (CRP). Although no changes were seen in the cytokine responses to the supplementation intervention at baseline, it was noted that the whey protein (WP) group experienced a decrease in plasma IL-1α and IL-1β following the intervention (week zero: IL-1α = 28.9 ± 8.9 pg/mL, IL-1β = 61.5 ± 7.9 pg/mL; week six: IL-1α = 23.1 ± 6.6, IL-1β = 55.5 ± 4.7), when compared with the soy protein group (week zero: IL-1α = 14.9 ± 4.2 pg/mL, IL-1β = 57.0 ± 5.6 pg/mL; week six: IL-1α = 13.0 ± 6.7, IL-1β = 58.9 ± 6.7). In addition to this finding, it was noted that neither the whey or soy protein interventions has significant effects on plasma concentrations of IL-2, IL-6, IL-8, TNF-α, or IFN-γ at week six of supplementation. Furthermore, a slight decrease in CRP was found following the six-week program with the WP group, however, this decrease was not significant (WP: week zero = 3.1 ± 1.0 mg/L; week six = 2.4 ± 0.6 mg/L; vs. soy protein group: week zero = 1.7 ± 0.3 mg/L; week six = 1.8 ± 0.4 mg/L). 

Following 300 strenuous eccentric contractions, both blueberry consumption and the consumption of a control beverage inflammatory markers (IL-6 and CK) were elevated [25]. Inflammatory markers showed increases following the damaging exercise bout. Creatine kinase elicited a gradual and significant increase in both conditions between pre- and 36 h post-damage (*p* < 0.05); however, no interaction effect was observed between time and treatment (*p* = 0.426). Plasma IL-6 showed a similar trend, with a gradual increase in the inflammatory marker occurring following the damaging exercise bout. McLeay, et al. [25] noted a significant difference (*p* < 0.05) between the pre-damaging levels, and following 36 and 60 h of recovery in both the blueberry and control conditions. No blueberry treatment (*p* = 0.198) or time x treatment interactions (*p* = 0.721) were detected. Although increases in biomarkers of muscle damage are evident following a bout of muscle damaging exercise, supplementation with whey protein appears to reduce the response of some of these markers, namely IL-1α, IL-1β and CRP, indicating its capability of enhancing recovery, non-significantly though.

#### 3.2.4. Oxidative Stress and Anti-Oxidative Markers

Oxidative stress is described as a disturbance in the balance between the production of reactive oxygen species (free radicals) and antioxidant defences [63]. Two of the selected studies examined the effects of supplementation interventions on markers of oxidative stress. 

Oxidative stress (ROS-generating potential and protein carbonyls) was shown to increase following 300 eccentric contractions in a study conducted by McLeay, et al. [25]. Significant increases (*p* < 0.01) in plasma oxidative stress, ROS-generating potential and protein carbonyls were detected 12 h post-damaging exercise in both conditions. Following 36 h post-damage, a gradual decline in ROS-generating potential was noted in the blueberry condition, while it remained elevated in the control condition (*p* < 0.01). With regards to the protein carbonyls, a large and significant increase (*p* < 0.01) was identified at 12 h in both conditions. An accelerated decrease in protein carbonyls was observed in the blueberry group following the 12 h mark, however, this decrease was not significant (*p* = 0.06).

Within the same study, total anti-oxidant capacity (TAC) was measured, showing no significant impact of blueberry consumption (*p* = 0.149) on plasma anti-oxidant capacity prior to the participants performing the 300 eccentric contractions in comparison to the control (*p* = 0.140). When comparing the pre-damage measurements with those taken at 60 h post-damage, a significant treatment x time interaction (*p* = 0.038) was observed. McLeay, et al. [25] stated that this interaction indicates that the consumption of blueberries improved the plasma TAC levels following strenuous eccentric exercise.

Tara and colleagues [59] analyzed oxidative damage, and noted a significantly decreased thiobarbituric acid reactive substances (TBARS) response at week six (1.0 ± 0.2 μmol/L) in the WP group, when compared with week zero (1.7 ± 0.2 μmol/L, *p* < 0.05); while the soy protein group showed no effect on TBARS over the study period (week zero: 1.3 ± 0.2 μmol/L, week six: 1.4 ± 0.2 μmol/L) [59]. Furthermore, protein carbonyl concentrations were not significantly different at baseline or at week 6 in the treatment groups (week zero: WP group = 7.0 ± 0.6 mmol/L, soy protein group = 6.4 ± 0.6 mmol/L; week six: WP group = 7.1 ± 0.6 mmol/L, soy protein group = 8.1 ± 0.5 mmol/L). With regards to anti-oxidative activity, the authors noted no difference in plasma total anti-oxidative capacity at baseline or week six of the study. Reduced glutathione (GSH) concentrations showed no difference at baseline between treatment groups, while the soy protein group showed decreased GSH concentrations at week six compared to baseline (week zero: 1220 ± 70 μmo/L; week six: 1037 ± 36 μmol/L, *p* < 0.05), indicating reduced anti-oxidative activity, and no effect of WP ingestion was noted. The authors further commented that ingestion of WP could have a potential anti-oxidative action; despite this, their results demonstrated no improvements in anti-oxidant status with consumption of either soy protein or whey protein.

### 3.3. Sex-Related Differences with Supplementation Interventions

Reports of sex-related differences in EIMD are evident [6,30,41,54,64,65]. Although it has been reported that males and females may show similar decrements associated with EIMD, it has been noted that females tend to require longer periods of recovery for strength and muscular thickness, when compared to males following 8 sets of 10 concentric and eccentric repetitions of elbow flexion; however, the exact reason why this occurs is unclear [29]. On the other hand, Keane, et al. [30] noted that females may recover faster than males, as they are less fatigable than their male counterparts. With regards to muscle force loss following eccentric exercise, Goldfarb, et al. [66] note that similar losses should be experienced in both men and women. 

Recent investigation has indicated that female participation in sport and exercise research is under-presented. Costello, et al. [45] noted that females only represented 16%–36% of studies that investigated the management of EIMD and DOMS. This was further noted by Glenn, et al. [67] that females have been understudied within human research. Insufficient research in females has further been noted with regards to the response and effects of EIMD as well as supplementation [30,68].

## 4. Limitations

An important limitation is that not all the studies included in this review made mention of the phase of the menstrual cycle during which the female participants were tested. One study mentioned the testing occurring during the luteal phase [25], with another indicating that the majority of the participants (11/12) began testing in the follicular phase, with five of these participants remaining in this phase for the duration of the study, and six transitioning to the luteal phase; the remaining participant completed the study in the luteal phase. Although Green et al. (2008) did indicate that the menstrual cycle phase did not have an effect on the dependent variables, it has been indicated in other studies that the menstrual cycle phase could influence the amount of muscle damage experienced by the female participants [6,35,37,48,64,69,70]. It has been reported that oestrogen has the ability to influence skeletal muscle growth, gene expression, metabolism, contraction characteristics, and maintain muscle mass [34,35,71]. Tiidus [34] stated that resting serum CK levels in females correspond inversely with levels of oestrogen, indicating that the time of testing in relation to the phases of the menstrual cycle is important. Moreover, the phase of menstrual cycle and it’s corresponding level of oestrogen, as well as oral contraceptive use has been shown to impact the physiological response to exercise [33].

An additional limitation is that it is possible that not all relevant papers were retrieved. Excluded keywords may have eliminated relevant papers not identified from searching reference lists of retrieved papers.

## 5. Conclusions and Recommendations

Blueberry and Protein (whey and casein) supplementation interventions in females appear to improve muscle performance indicators [25,58,62], muscle protein synthesis or have an effect on the extent of muscle damage experienced, both perceived and measured using biomarkers as indicators of muscle damage and inflammation [59]. It is evident from the studies brought forward in this review that the type and timing of supplementation strategy employed will determine the effects that the supplementation intervention strategy would have on exercise-induced muscle damage. Research suggests that some markers of muscle damage and recovery do not differ between sexes. The conflicting evidence [30,31,66] surrounding sex-related differences in response to exercise makes it difficult to deduce from studies completed in males, to females regarding the effects that nutritional intervention strategies may have in attenuating the effects of EIMD. Recent investigation has indicated that female participation in sport and exercise research is under-presented. Costello, et al. [45] noted that females only represented 16%–36% of studies that investigated the management of EIMD and DOMS while insufficient research on females has been conducted with regards to the response and effects of EIMD as well as supplementation [30,68]. In general, Glenn, et al. [67] reported that females have been understudied within human research. It is therefore warranted that further research be conducted, both in recreationally active and elite athletes, in order to better understand the effects of supplementation intervention strategies on recovery for female athletes. As well as comparing the different timings of supplementation, pre-, during, or post-exercise. Furthermore, it is suggested that the future research be conducted during the different menstrual cycle phases, as well as taking contraception method into account. Moreover, these studies should include a male group, in order to allow for cross-examination of the effects of supplementation. Moreover, as evident from the studies mentioned, supplementation with tart cherries and pomegranate, among other foods, may be a good alternative to other supplements. 

The diversity of the methodologies used to assess the efficacy of supplementation interventions indicates the importance of providing a clear logical progression through the different aspects of supplementation in order to produce a clear concise set of criteria for its efficacy. The timing of supplementation, as well as when during the menstrual cycle the supplementation and exercise interventions occurred are of importance to further understand the efficacy of the supplementation intervention. 

## Figures and Tables

**Table 1:** A summary of the studies included in the review.

Reference	Participants	Exercise Protocol	Supplementation Protocol	Time of Supplementation	Study Design
Green, et al. [60]	18 females (age: 24.6 ± 3.3 years) Low to moderate level of aerobic activity (1–5 h/week)	Intermittent 30 min Downhill Run (−12% grade at 8.0 mph) Six intervals of 5 min each with 2-min standing rest between intervals	*Hour 1 Post-Ex.:* CHO—1.2 g CHO/kg Body weight (BW) CHO-Protein—1.2 g CHO/kg BW + 0.3 g Protein/kg BW Placebo—noncaloric, artificially sweetened drink, isovolumetric to CHO and CHO + protein beverages *Hour 2 Post-Ex.:* CHO—0.6 g CHO/kg body weight (BW) CHO-Protein—0.6 g CHO/kg BW + 0.15 g Protein/kg BW Placebo—noncaloric, artificially sweetened drink, isovolumetric to CHO and CHO + protein beverages	Post-exercise	Double blind, placebo controlled, non-crossover study
McLeay, et al. [25]	10 healthy, resistance trained physically active females (age: 22 ± 1 year) (participating in recreational resistance and aerobic exercise at least twice a week)	Three sets of 100 eccentric repetitions of quadriceps on an isokinetic dynamometer Passive recovery lasting 5 min between sets	Blueberry beverage: 200 g frozen New Zealand blueberries blended with 1 banana (approx. 50 g) and 200 mL commercial apple juice Control beverage: 25 g dextrose blended with 1 banana (approx. 50 g) and 200 mL commercial apple juice	Immediately post (evening), and 12 and 36 h post (morning). No treatment taken at 60 h post	Randomized, balanced, cross-over study
Tara, et al. [59]	18 healthy endurance female athletes (age: 21.3 ± 0.4 years)	Minimum of 1 h running per week, maintain normal training routine	Whey protein isolate—89.3 g protein, 1.1 g fat, 3.6 g CHO per 100 g Soy protein isolate—83.3 g protein, 4.2 g fat, <2.1 g CHO, 175 mg isoflavones per 100 g Each serving standardized to 40 g protein consumed daily as a drink, for 6 weeks	No specified time point	Randomized, double-blind
Wilborn, et al. [58]	16 female resistance trained basketball players (age: WP 20.0 ± 1.9 years; CP 21.0 ± 2.8 years)	Periodised anaerobic resistance-training program 4 days per week for 8 weeks. 2 upper- and 2 lower-extremity workouts per week. 30–40 min skill and conditioning related work	Whey protein (WP) group—24 g Optimum nutrition 100% whey gold standard protein (120 Cal, 1 g fat, 4 g CHO, 24 g Protein) Casein protein (CP) group—24 g Optimum nutrition 100% casein protein (120 Cal, 1 g fat, 3 g CHO, 24 g protein) 30 min before and immediately after each training session for 8 weeks. Protein mixed with 10fl oz water	Pre- and post-exercise	Randomized, double-blind
Taylor, et al. [62]	14 NCAA Division III Female Basketball Players (WP: age, 20 ± 2 years; MD: age, 21 ± 3 years)	8 week resistance training: 4 days/week undulating aerobic and resistance-training (3 days/week = resistance-training; 3 days/week = “explosive” exercises; 4 days/week = agility and conditioning)	Immediately prior to and following supervised workouts, for 8 weeks, dissolved in water: 24 g WP—1951 kcal/day, 92 ± 6 g/day protein, 263 ± 30 g/day CHO, 64 ± 6 g/day fat 24 g maltodextrin (MD)—1875 kcal/day, 74 ± 12 g/day protein, 283 ± 34 g CHO, 75 ± 17 g/day fat	Immediately prior to and following resistance training workout	Matched according to DEXA lean muscle mass, double blind and randomly assigned to supplement

**Table 2:** Results of muscle damage and inflammatory markers in response to supplementation intervention strategies (* indicating significant changes).

Reference	Markers of Muscle Damage and Inflammation	Result	Difference Among Groups
Green et al. [60]	Serum creatine kinase (CK) activity	Small ↑ immediately post DHR Peak 1 day post DHR ↓ Day 2 and 3 post DHR	CHO group greater ↑ in CK compared to both the CHO/protein and placebo groups
Tara et al. [59]	CRP	Slight ↓ following 6 week program (whey)	Whey protein supplement ↓ CRP at week 6 vs. soy protein
	IL-1α	No significant differences following 6 week program	Whey protein ↓ plasma IL-1α and IL-1β post-intervention vs. soy protein
	IL-1β		No difference between groups (whey and soy protein groups)
	IL-2		
	IL-6		
	IL-8		
	TNF-α		
	IFN-γ		
	IL-6	↑ post-exercise	PRO ↓ IL-6 response to exercise compared with placebo
McLeay, et al. [25]	ROS-generating potential	↑ 12 h post-exercise and gradually decreased at 36 h	Blueberry condition ↓ ROS-generating potential post, compared to control
	Protein carbonyls	↑ post-exercise, followed by gradual ↓	Accelerated ↓ in blueberry condition following 12 h post-exercise in comparison to the control
	CK	↑ post-exercise	CK lower in blueberry condition at 60 h post vs. Control condition
	IL-6	↑ post-exercise	No significant difference between conditions
	Total anti-oxidant capacity (TAC)	↑ post-exercise	Blueberry consumption increased plasma anti-oxidant capacity
